# Supplementary material for: Design of stretchable and self-powered sensing device for portable and remote trace biomarkers detection
Source: Nat Commun. 2023 Aug 26;14:5221. doi: 10.1038/s41467-023-40953-z (PMC10460451; doi:10.1038/s41467-023-40953-z)
Supplement: Supplementary file 1 — Supplementary Information [file 41467_2023_40953_MOESM1_ESM.pdf]

Supplementary Information

Design of stretchable and self-powered sensing device for portable and remote trace biomarkers detection

Wenxi Huang[1],† Qiongling Ding[1],† Hao Wang[1],† Zixuan Wu[1], Yibing Luo[1], Wenxiong Shi[2], Le Yang[3], Yujie Liang[4], Chuan Liu[1], and Jin Wu[1]*

[1]State Key Laboratory of Optoelectronic Materials and Technologies, Guangdong Province Key Laboratory of Display Material and Technology, School of Electronics and Information Technology, Sun Yat-sen University, Guangzhou 510275, China. [2]Institute for New Energy Materials and Low Carbon Technologies, School of Materials Science and Engineering, Tianjin University of Technology, Tianjin 300384, China. [3]Guanghua School of Stomatology, Hospital of Stomatology, Sun Yat-sen University, 56th Lingyuanxi Road, Guangzhou, Guangdong, 510055, China; Guangdong Province Key Laboratory of Stomatology, No. 74, 2nd Zhongshan Road, Guangzhou, 510080, Guangdong, China. [4]Department of Oral and Maxillofacial Surgery, Guanghua School of Stomatology, Hospital of Stomatology, Sun Yat-sen University, 56th Lingyuanxi Road, Guangzhou, Guangdong, 510055, China; Guangdong Province Key Laboratory of Stomatology, No. 74, 2nd Zhongshan Road, Guangzhou, 510080, Guangdong, China. *email: wujin8@mail.sysu.edu.cn

†These authors contributed equally to this work.

Supplementary Figures

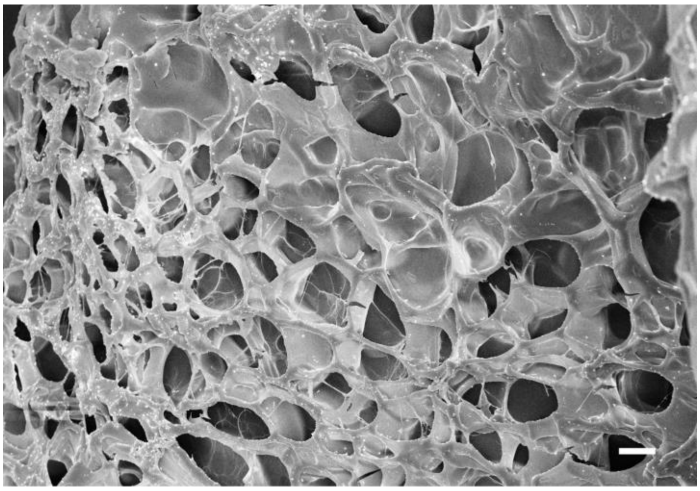

Supplementary Figure 1. Scanning electron microscopy (SEM) image of double network (DN) hydrogel. The scale bar is 10 μm. The experiment was repeated 3 times independently with similar results.

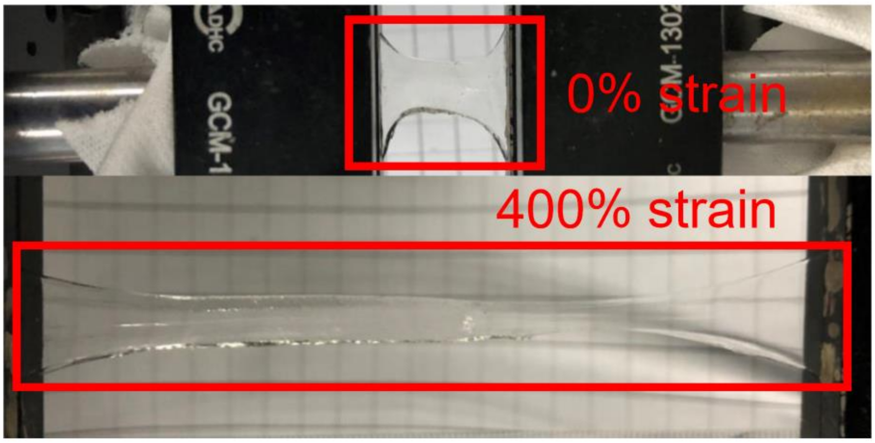

Supplementary Figure 2. Photographs of pristine DN hydrogel (DNH) at 0% and 400% tensile strains.

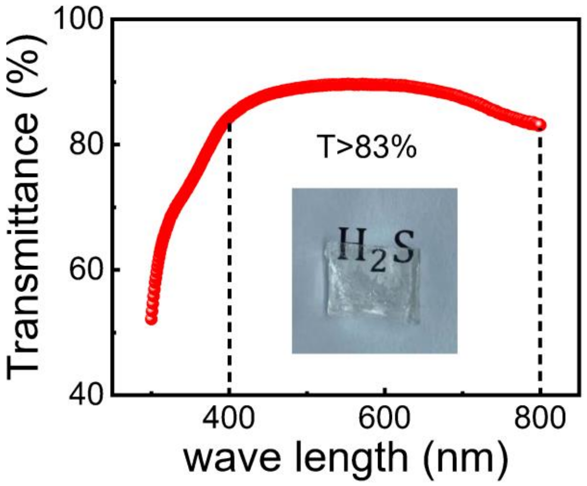

Supplementary Figure 3. Transmittance spectra of pristine DNH. Inset is the photograph of a piece of pristine DNH placed on a word “H_2S”.

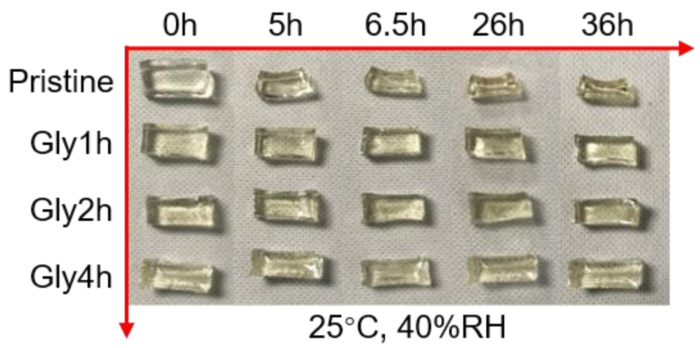

Supplementary Figure 4. Photographs showing the morphology evolutions of pristine DNH and organohydrogels (DNOs) when stored at 25°C, 40% relative humidity (RH) for a series of time.

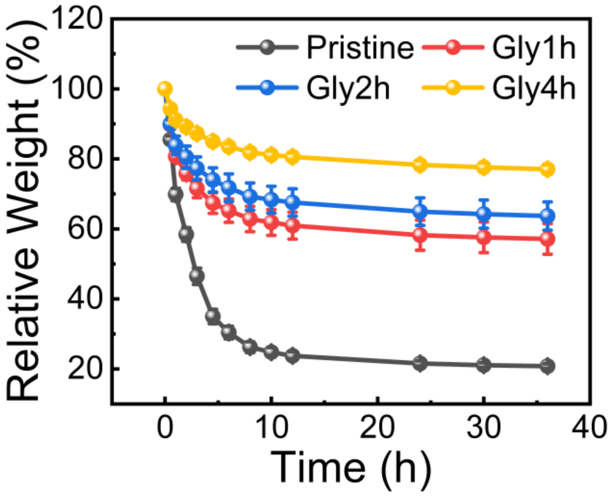

Supplementary Figure 5. Relative weight change of pristine DNHs and DNOs when stored at 25°C, 40% RH for a series of time. n = 3 for each group. The error bars denote standard deviations of the mean.

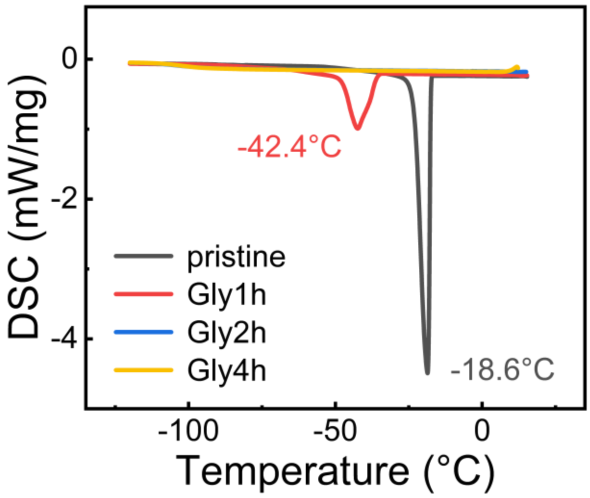

Supplementary Figure 6. Differential scanning calorimetry (DSC) spectra of pristine DNH and DNOs obtained by soaking in glycerol (Gly) for different time.

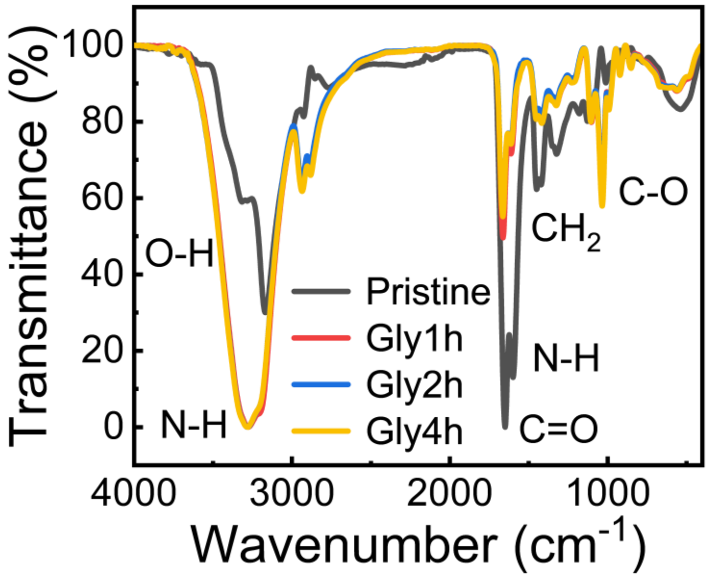

Supplementary Figure 7. Fourier transform-infrared (FTIR) spectra of pristine DNH and DNOs obtained by soaking in Gly for different time.

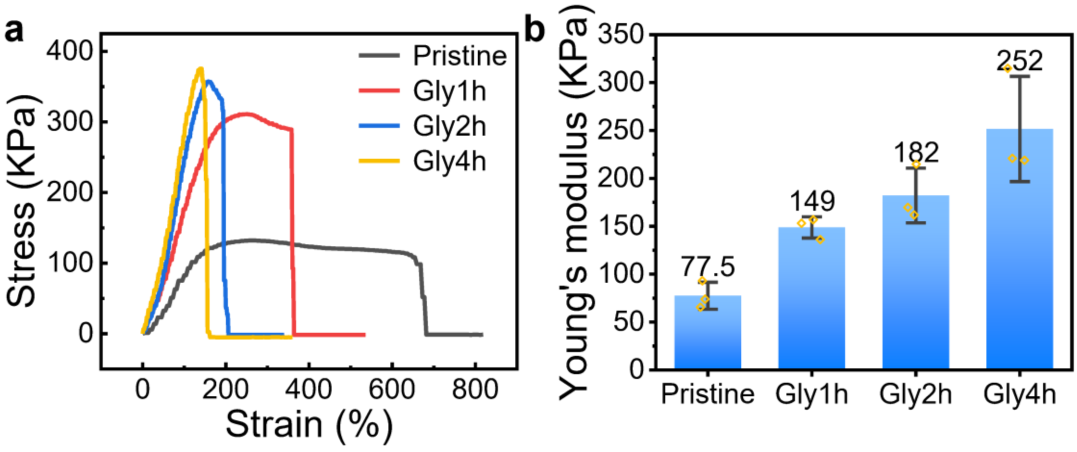

Supplementary Figure 8. Mechanical properties of pristine DNH and DNOs obtained by soaking in Gly for different time. a stress-strain curves; **b** Young's modulus. n = 3 for each group. The error bars denote standard deviations of the mean.

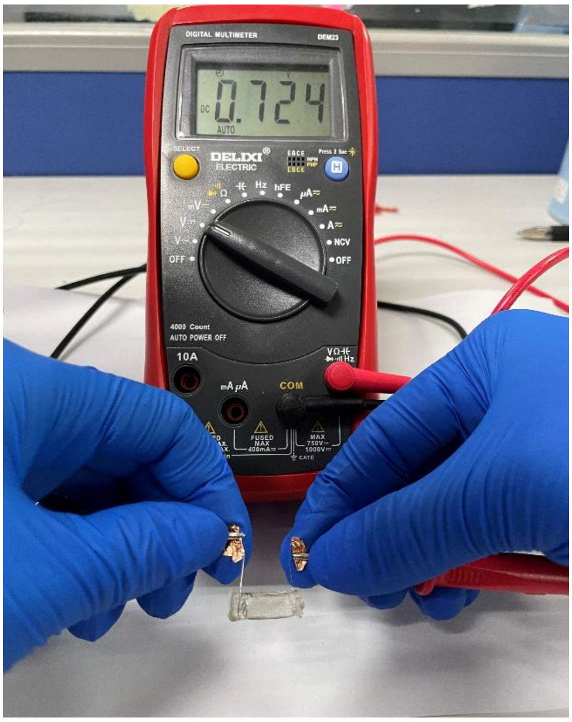

Supplementary Figure 9. A physical picture of the self-powered sensor (Zn/Ag/DNH).

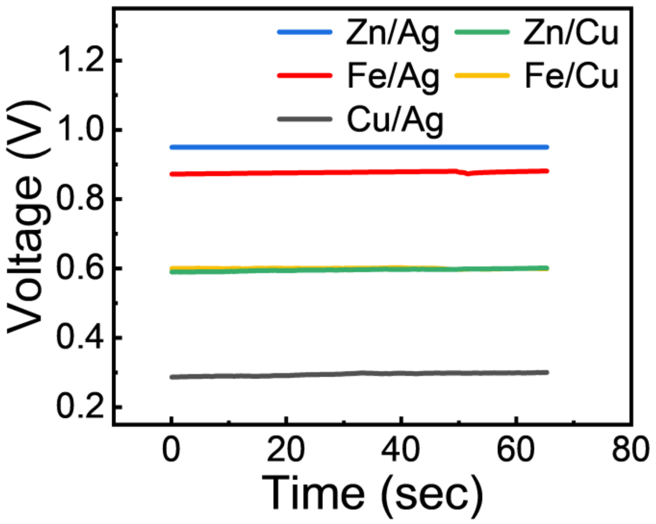

Supplementary Figure 10. **OCV of battery-sensor complexes with different metal electrodes.**

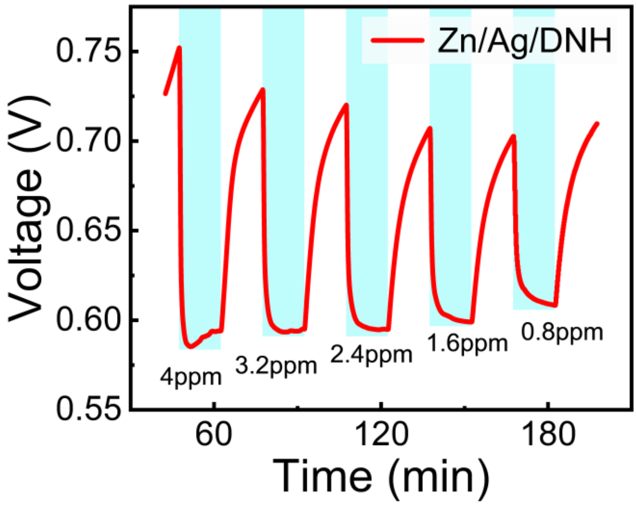

Supplementary Figure 11. Dynamic change in the Open-circuit voltage (OCV) of the device with Zn-DNH-Ag structure to H_2S gas with reduced concentration from 4 to 0.8 ppm.

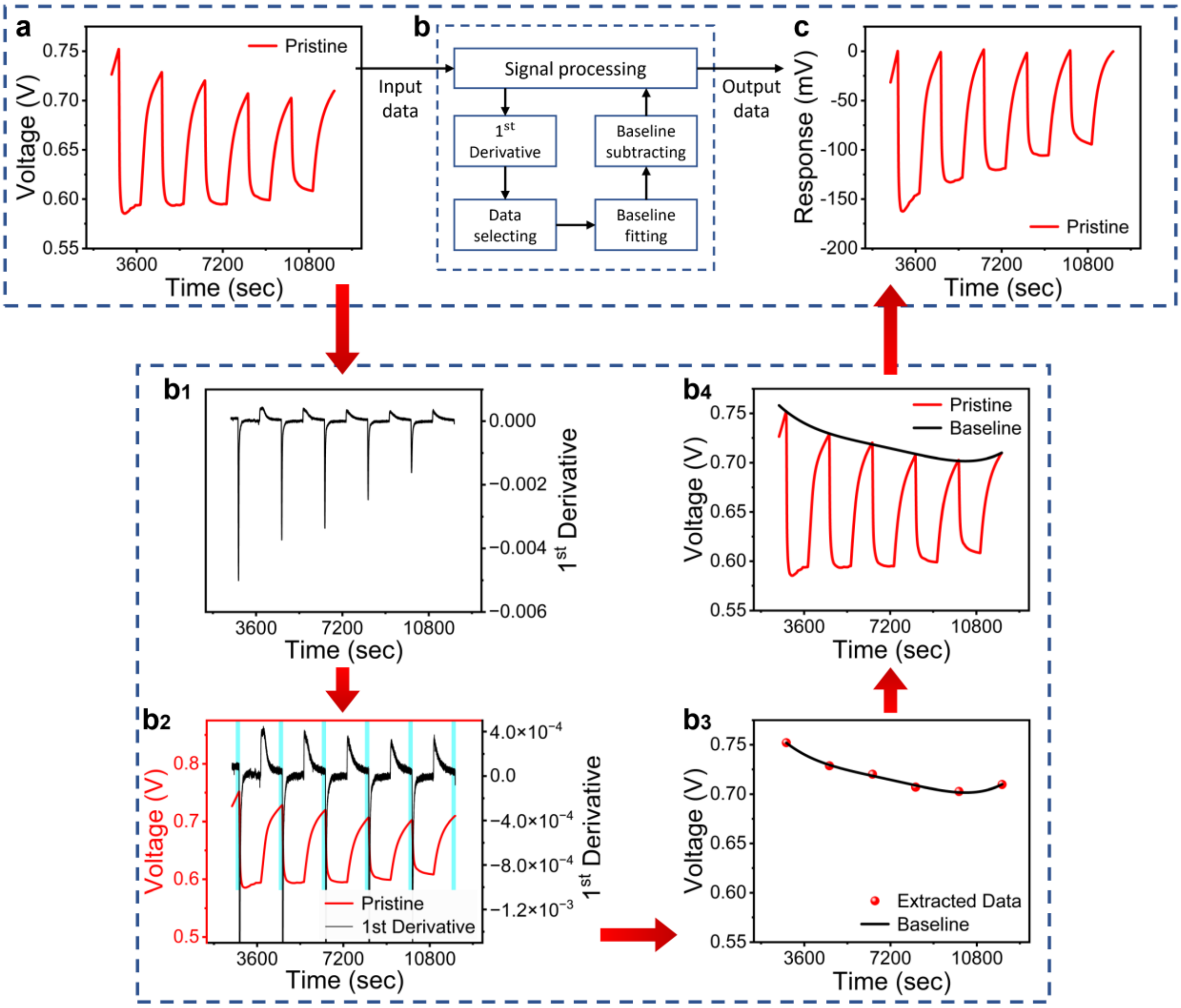

Supplementary Figure 12. Baseline correction. a The original OCV of Zn/Ag/DNH sensor to H_2S before baseline correction. **b** The procedure of baseline correction: **b₁** The 1st derivative curve of the voltage in **a**. **b₂** The original dynamic OCV and its 1st derivative curve of Zn/Ag/DNH sensor to H_2S. **b₃** The extracted data and corrected baseline of dynamic OCV. **b₄** The baseline correction of the dynamic OCV to H_2S. **c** Corrected response curve of Zn/Ag/DNH sensor to H_2S.

The process of baseline correction is as follow. Firstly, the first order derivative of the original OCV data was obtained (**b₁**). Secondly, the original data points whose corresponding first derivative values were located in the recovery period and were closest to 0 were selected (marked in cyan in **b₂**). Then, the extracted data were used for polynomial fitting to obtain the

baseline (**b**$_3$). Lastly, the corrected response curve was obtained by subtracting the baseline from the original dynamic OCV (**b**$_4$).

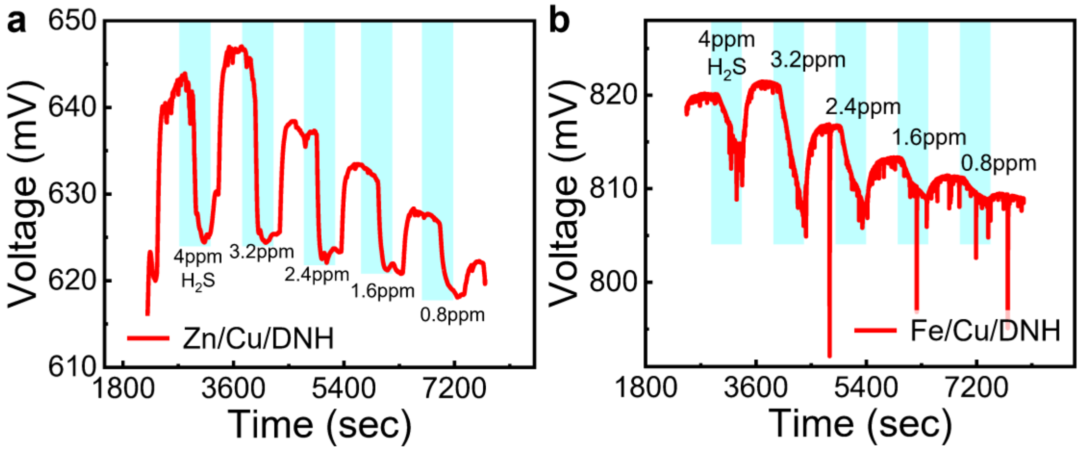

Supplementary Figure 13. Dynamic OCV change of different sensors made of different electrode materials to H_2S gas with reduced concentration from 4 to 0.8 ppm. a Zn/Cu/DNH; **b** Fe/Cu/DNH.

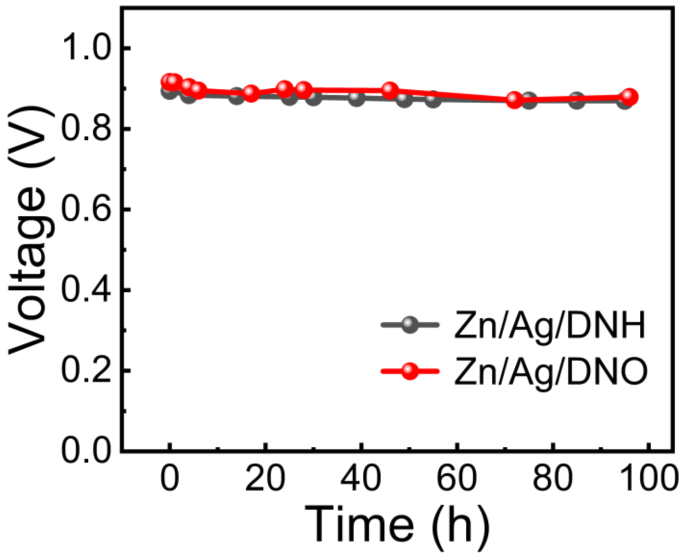

Supplementary Figure 14. OCVs of Zn/Ag/DNH and Zn/Ag/DNO sensors were intermittently recorded over 96 h, showing the stability of the OCV.

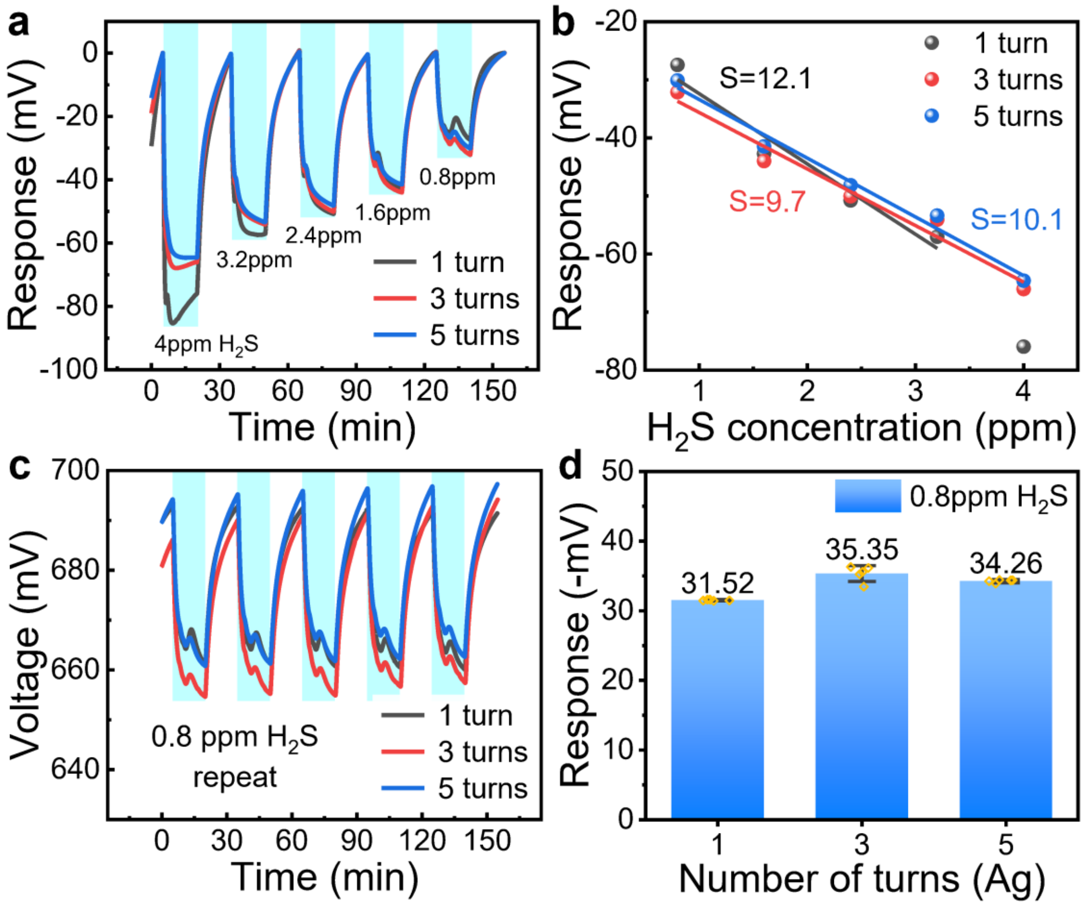

Supplementary Figure 15. H_2S sensing properties of the Zn/Ag/DNO sensor with different numbers of Ag wire turns (diameter: 0.05 mm). a Dynamic responses to H_2S gas with reduced concentration from 4 to 0.8 ppm after baseline corrections. **b** Sensitivity derived from data in **a**. **c** Dynamic OCV change of the sensor in cyclic tests of 0.8 ppm H_2S. **d** Response histogram extracted from **c**. n = 5 for each group. The error bars denote standard deviations of the mean.

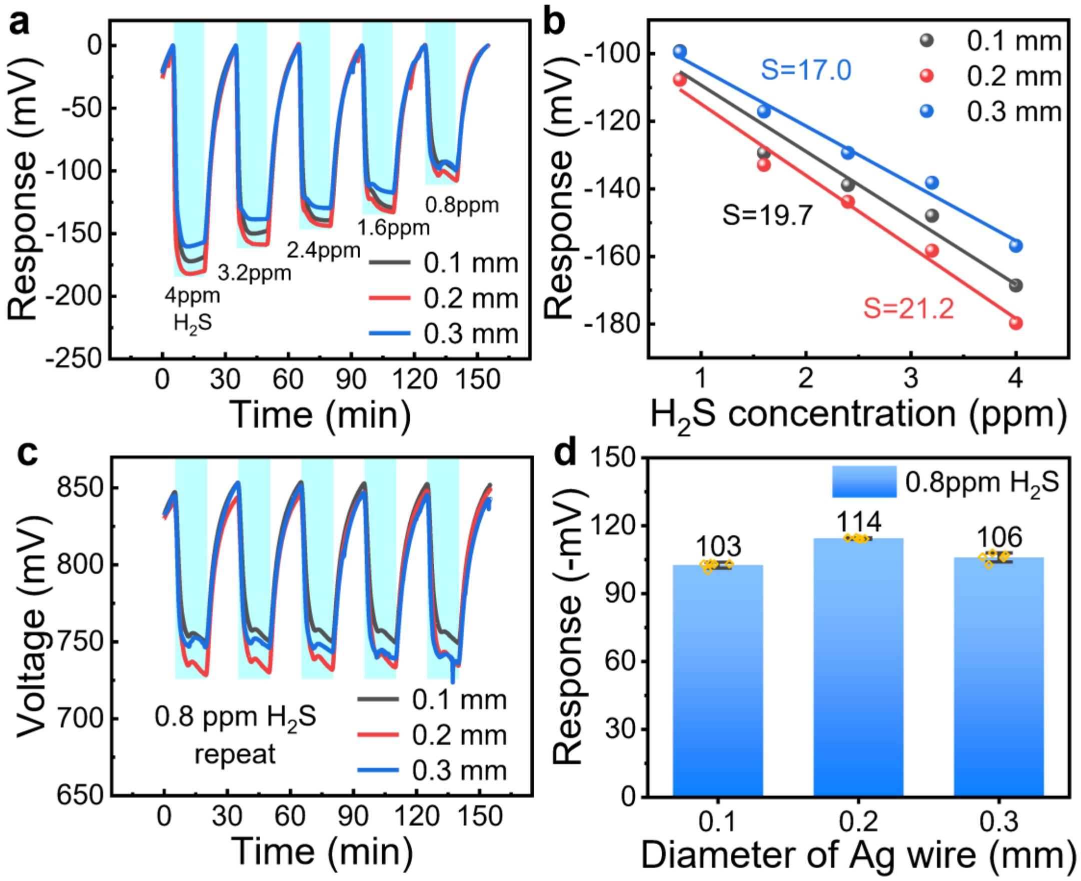

Supplementary Figure 16. H_2S sensing properties of the Zn/Ag/DNO sensor with different diameter of Ag wire. **a** Dynamic responses to H_2S gas with reduced concentration from 4 to 0.8 ppm after baseline corrections. **b** Sensitivity derived from data in **a**. **c** Dynamic OCV of the sensor in cyclic tests of 0.8 ppm H_2S. **d** Response histogram extracted from **c**. n = 5 for each group. The error bars denote standard deviations of the mean.

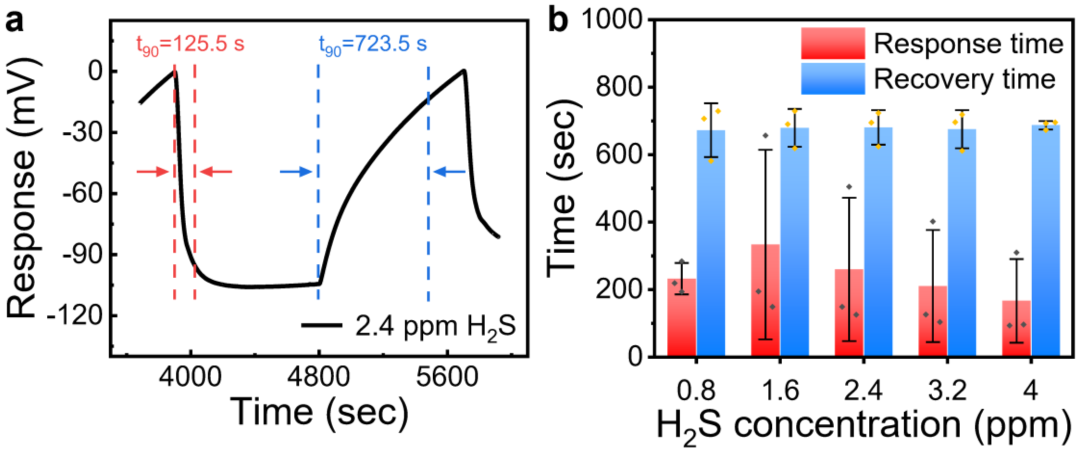

Supplementary Figure 17. Response/recovery time of Zn/Ag/DNO sensor. a Analysis of response/recovery time to 2.4 ppm H_2S. The curve is a segment extracted from Fig. 2c. **b** Response/recovery time of sensor to varying concentrations of H_2S. n = 3 for each group. The error bars denote standard deviations of the mean.

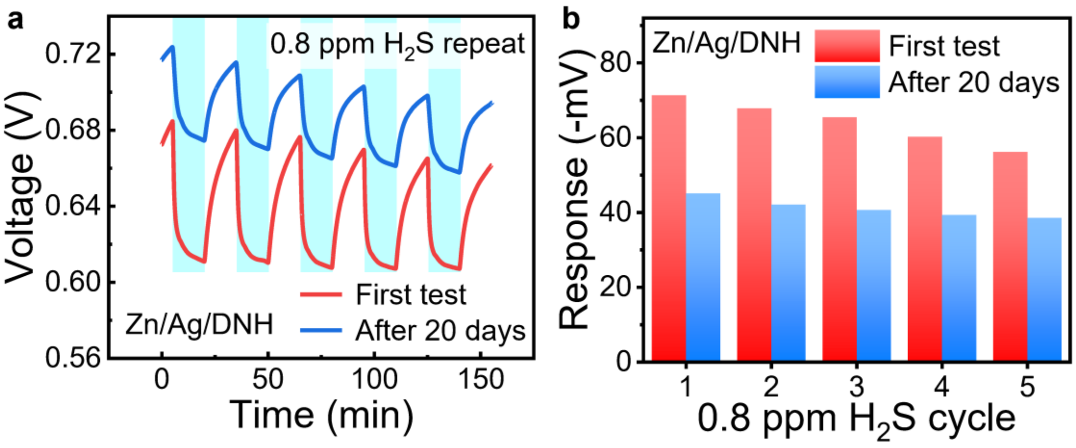

Supplementary Figure 18. Repeatability and stability of Zn/Ag/DNH sensor. a Dynamic OCV and **b** response histogram of Zn/Ag/DNH sensor to 0.8 ppm H_2S cycling for the first time and after 20 days of placement.

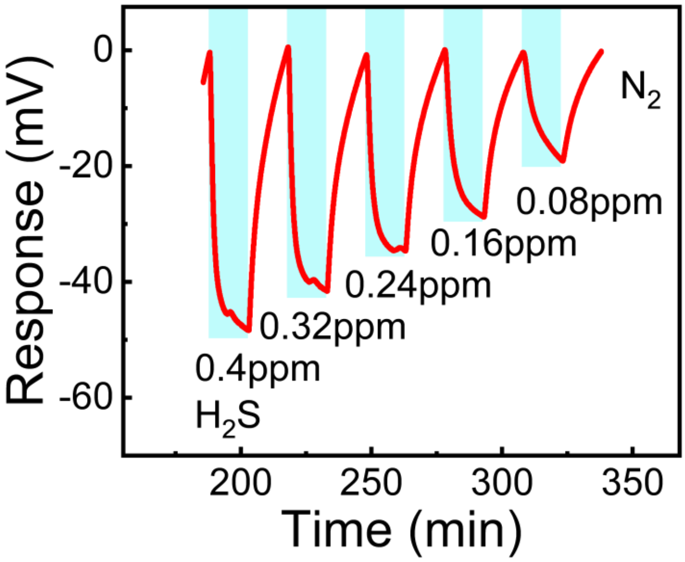

Supplementary Figure 19. Dynamic response curves of Zn/Ag/DNO to H_2S gas with relatively low concentrations (from 0.4 to 0.08 ppm).

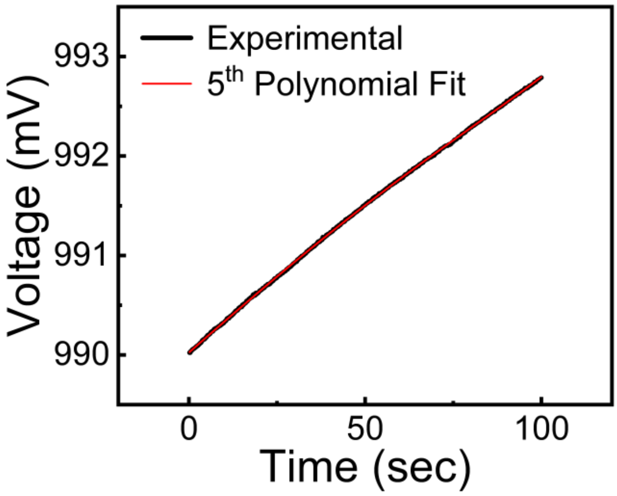

Supplementary Figure 20. Plots of the 5th order polynomial fitting graphs of OCV of the sensor at the baseline before exposure to H_2S, aiming at calculating the noise level.

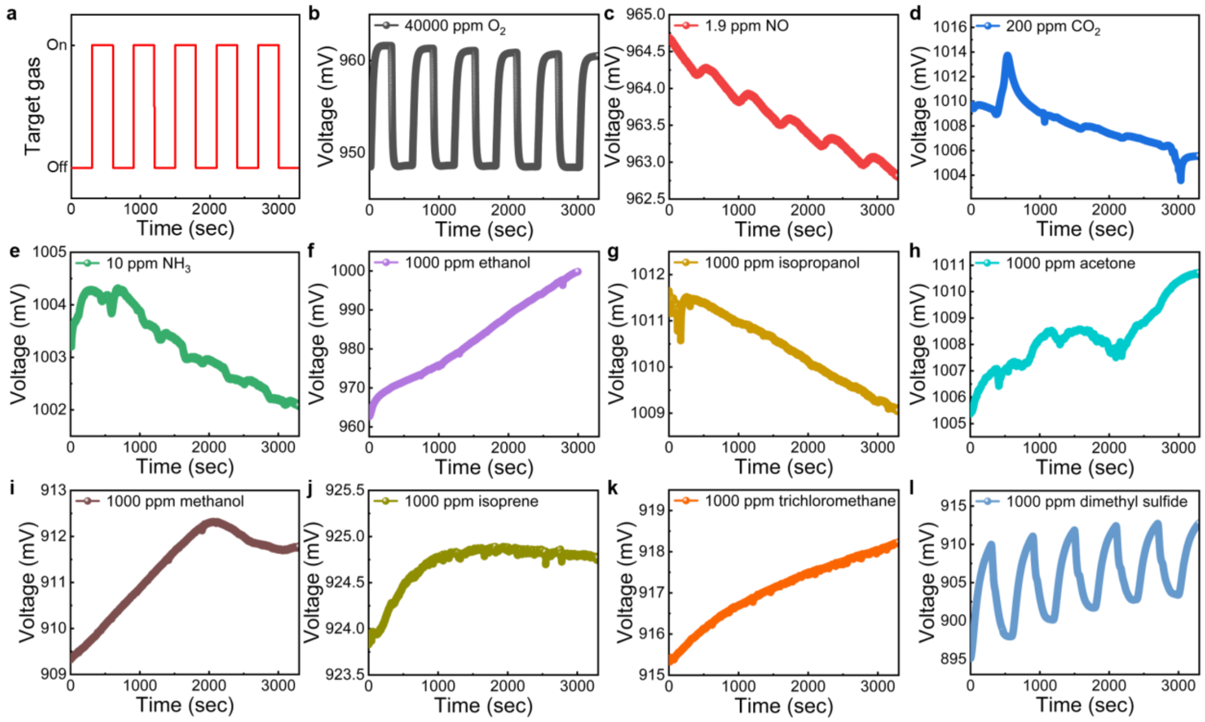

Supplementary Figure 21. Selectivity of Zn/Ag/DNO sensor. a Plot showing the process that controlled the periodic "on" and "off" of target gases to Zn/Ag/DNO sensor in (b-l). **b-l** OCV of the Zn/Ag/DNO sensor in the cyclic detection of **b** 40000 ppm O_2, **c** 1.9 ppm NO, **d** 200 ppm CO_2, **e** 10 ppm NH_3, **f** 1000 ppm ethanol, **g** 1000 ppm isopropanol, **h** 1000 ppm acetone, **i** 1000 ppm methanol, **j** 1000 ppm isoprene, **k** 1000 ppm trichloromethane, and **l** 1000 ppm dimethyl sulfide.

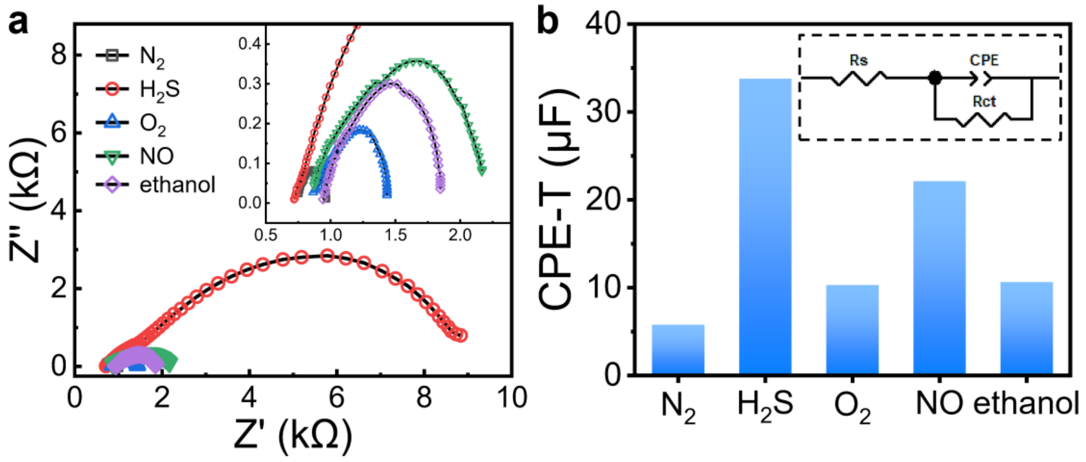

Supplementary Figure 22. Electrochemical impedance spectroscopy (EIS) analysis of the Zn/Ag/DNO sensor under different gas atmospheres. a Impedance spectra; b CPE-T values obtained by fitting. The insert is an equivalent circuit diagram of the Zn/Ag/DNO sensor.

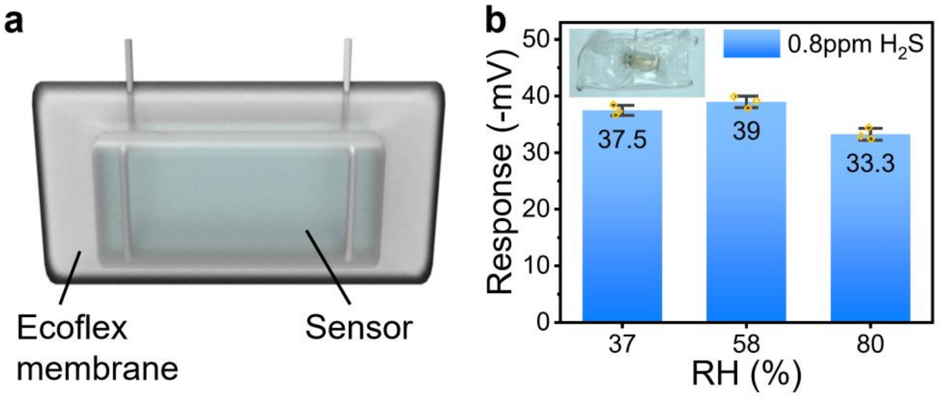

Supplementary Figure 23. Elimination of humidity interference on response. a Schematic diagram of Zn/Ag/DNO sensor with Ecoflex membrane encapsulation. **b** Response of the encapsulated sensor to 0.8 ppm H_2S under varying RH conditions (37%, 58%, 80%). Inset is a physical picture of the Zn/Ag/DNO sensor with Ecoflex membrane encapsulation. n = 3 for each group. The error bars denote standard deviations of the mean.

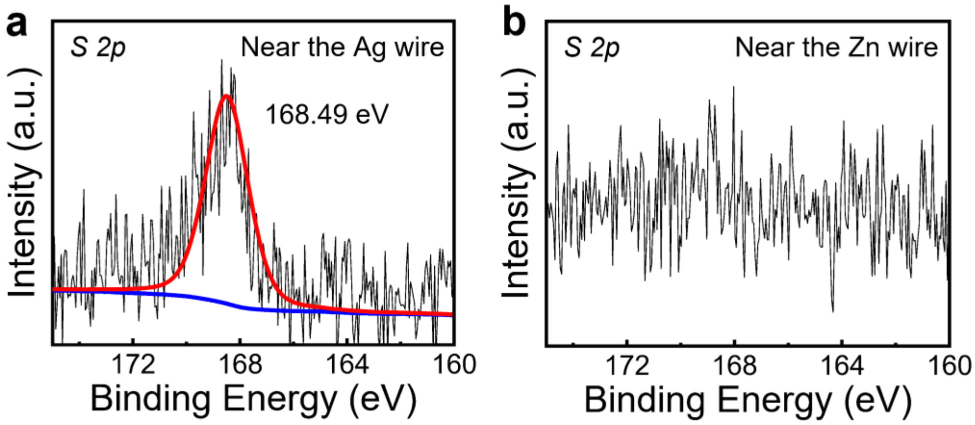

Supplementary Figure 24. X-ray photoelectron spectroscopy (XPS) analysis of hydrogel sample close to the electrodes after 10 h exposure to 2 ppm H_2S, showing *S 2p* data. a hydrogel sample near the Ag electrode; **b** hydrogel sample near the Ag electrode.

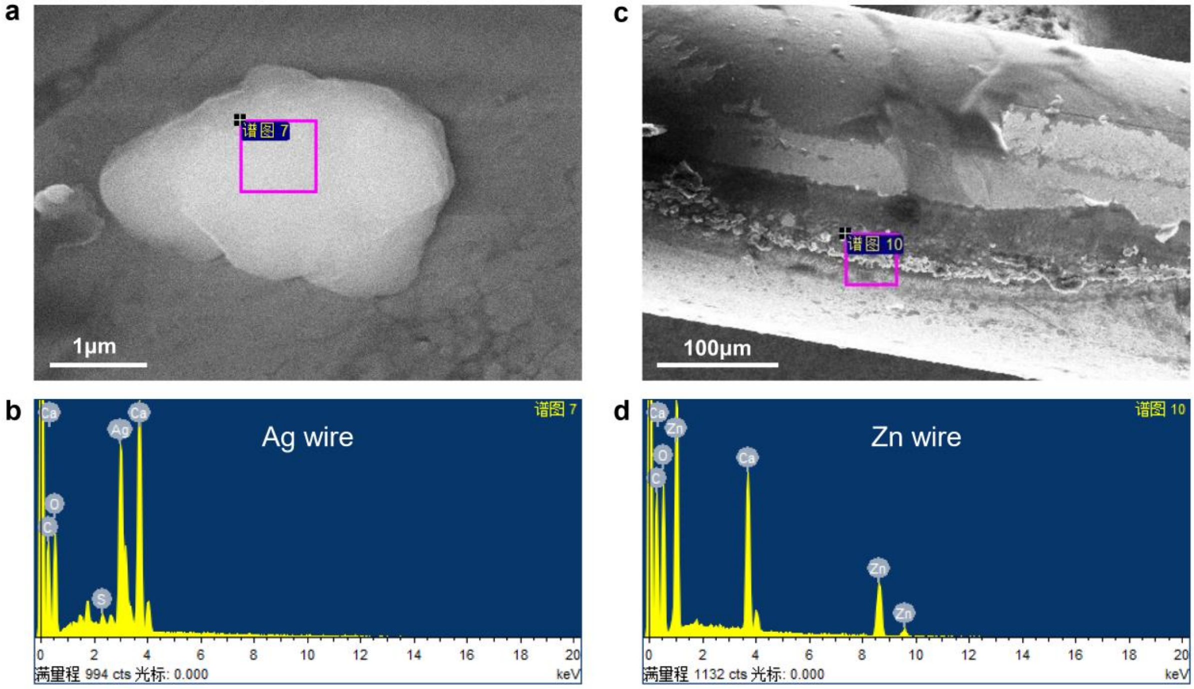

Element	Weight%	Atomic%
C K	17.25	30.33
O K	42.63	56.26
S K	0.43	0.29
Ca K	16.17	8.52
Ag L	23.51	4.60
Total	100.00	

Element	Weight%	Atomic%
C K	35.09	50.72
O K	37.21	40.38
Ca K	9.23	4.00
Zn K	18.48	4.91
Total	100.00	

Supplementary Figure 25. SEM images and Energy dispersive spectroscopy (EDS) element analysis of Ag and Zn wires from the device after exposure to H_2S for 10 h. a SEM image of Ag wire; **b** EDS of Ag wire; **c** SEM image of Zn wire; **d** EDS of Zn wire. The experiment was repeated 3 times independently with similar results.

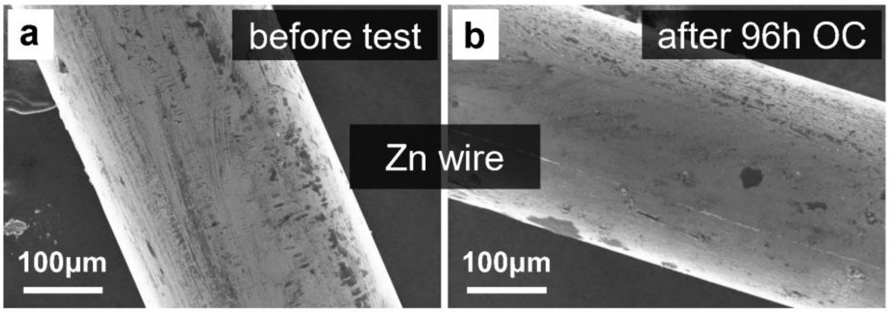

Supplementary Figure 26. SEM images before and after keeping the Zn/Ag/DNO in open-circuit (OC) state for 96 h. **a** Zn wire before test; **b** Zn wire after test. The experiment was repeated 3 times independently with similar results.

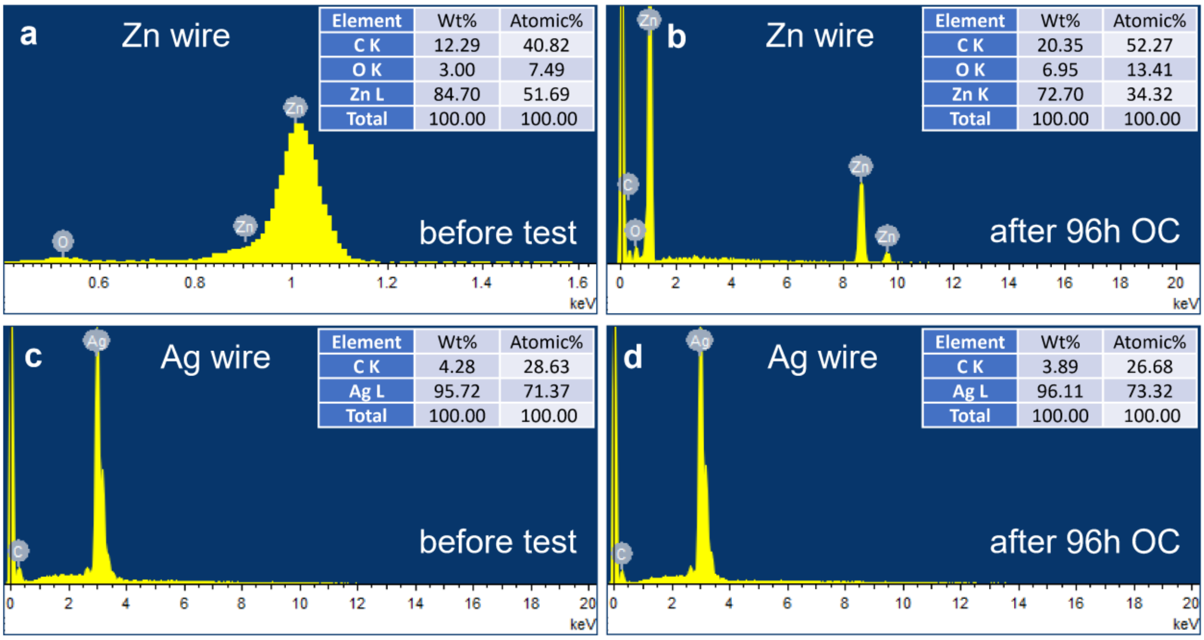

Supplementary Figure 27. EDS element analysis before and after keeping the Zn/Ag/DNO in OC state for 96 h. **a** Zn wire before test; **b** Zn wire after test; **c** Ag wire before test; **d** Ag wire after test.

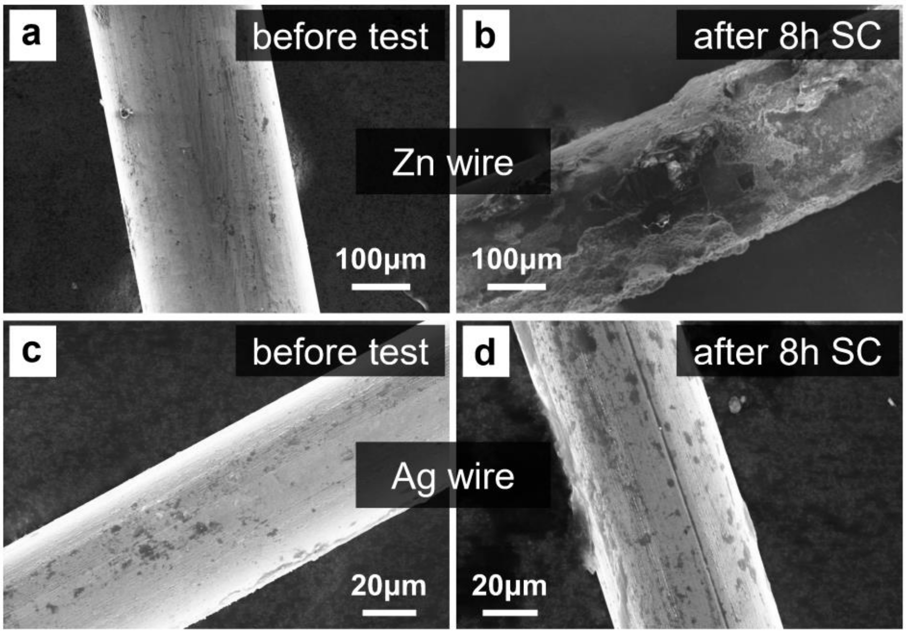

Supplementary Figure 28. SEM images before and after keeping the Zn/Ag/DNO in short-circuit (SC) state for 8 h. **a** Zn wire before test; **b** Zn wire after test; **c** Ag wire before test; **d** Ag wire after test. The experiment was repeated 3 times independently with similar results.

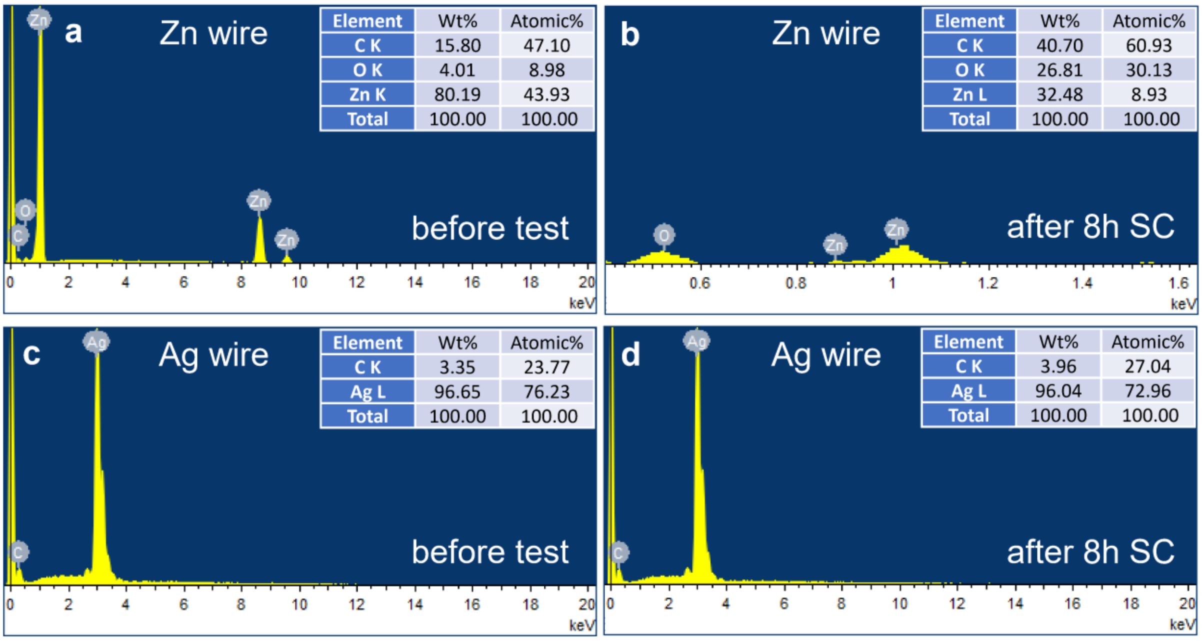

Supplementary Figure 29. EDS element analysis before and after keeping the Zn/Ag/DNO in SC state for 8 h. **a** Zn wire before test; **b** Zn wire after test; **c** Ag wire before test; **d** Ag wire after test.

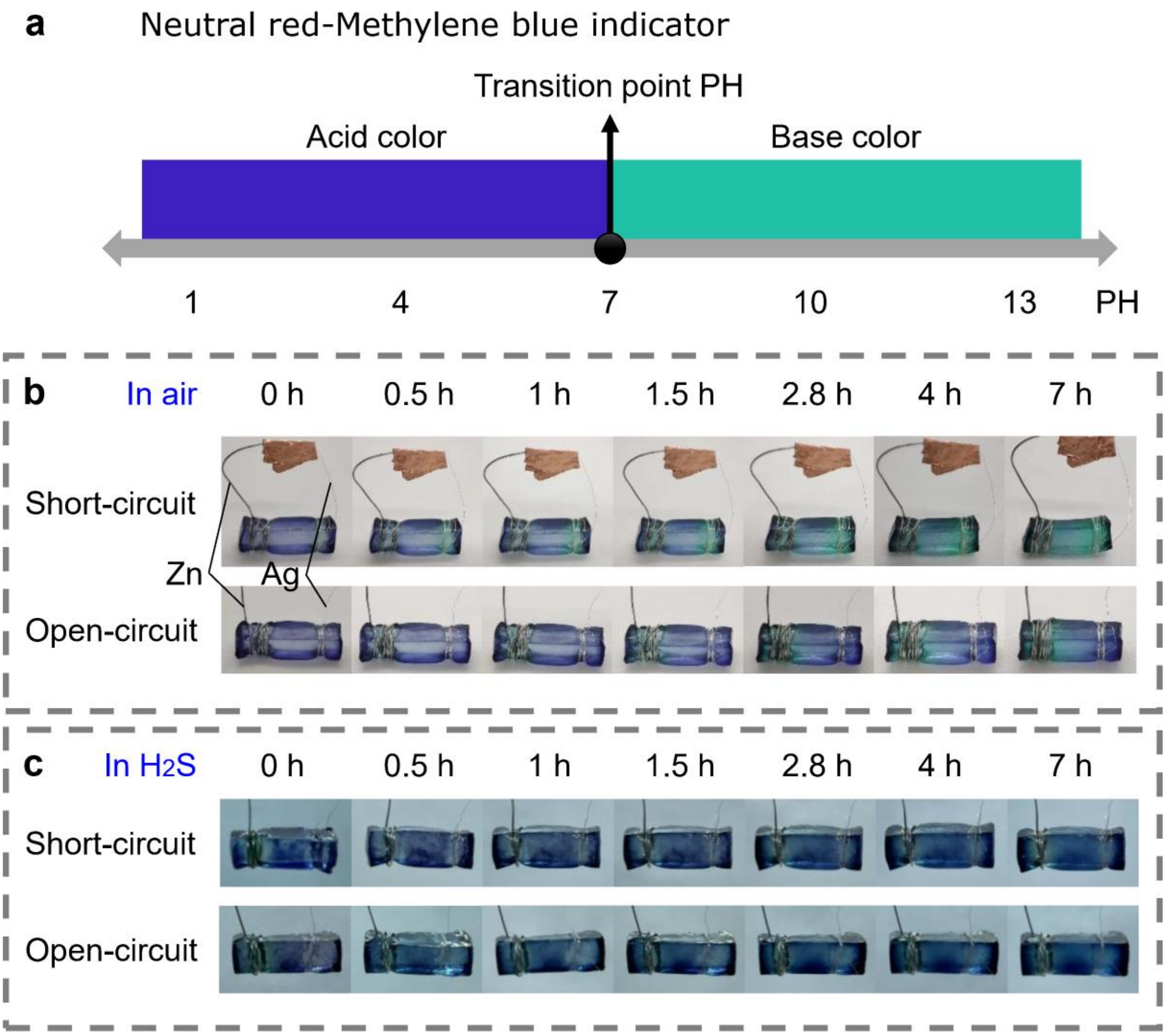

Supplementary Figure 30. PH changes of hydrogels near Zn (left) and Ag (right) electrodes explored by Neutral red-Methylene blue indicator. a The color of Neutral red-Methylene blue indicator corresponding to PH. **b-c** Photographs showing the color evolutions of Zn/Ag/DNHs after being stained with Neutral red-Methylene blue indicator and kept in SC state or OC state, in air (b) and H_2S (1 ppm) atmospheres (c).

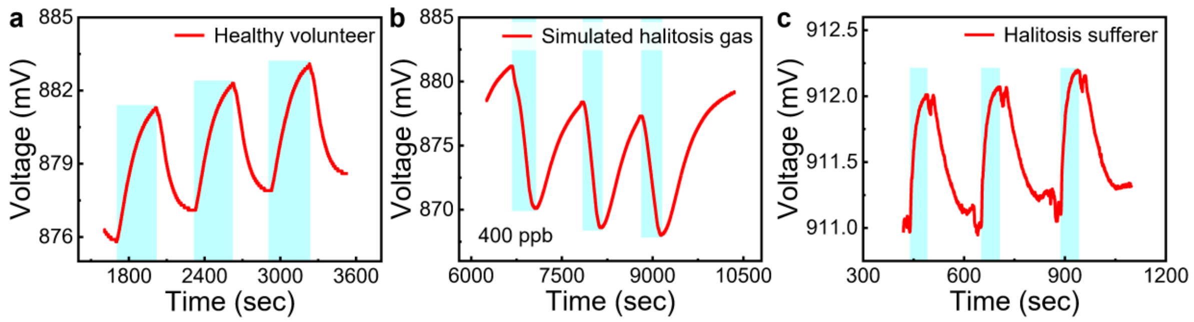

Supplementary Figure 31. Dynamic responses of the Zn/Ag/DNO sensor to the exhaled breath of a healthy volunteer/halitosis sufferer and simulated halitosis gas: a Exhaled breath of a healthy volunteer; **b** simulated halitosis gas; **c** Exhaled breath of a halitosis sufferer.

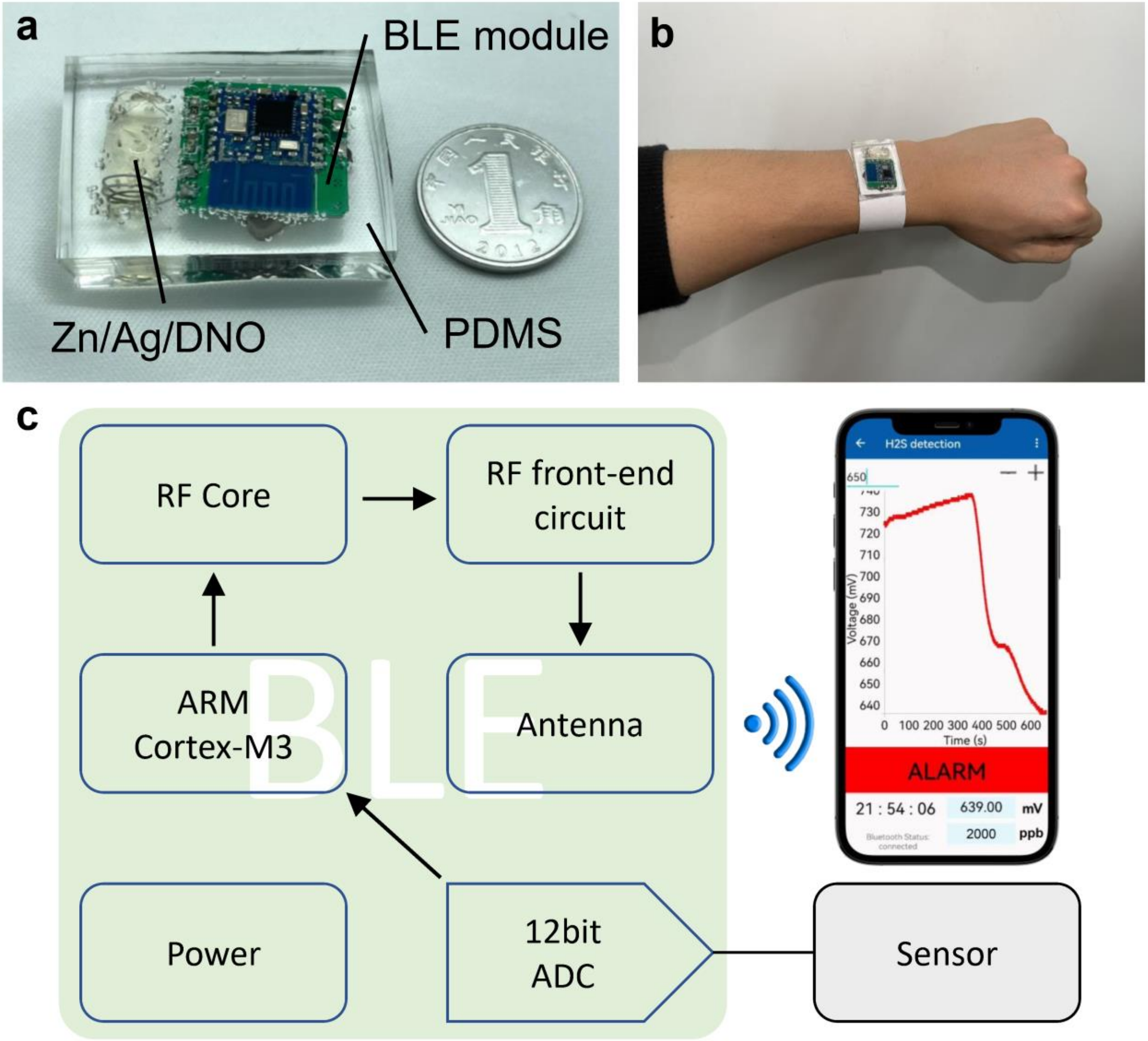

Supplementary Figure 32. Real photographs and schematic of the wireless H_2S sensing system. a Close-up of the sensing device. The size of a coin is larger than that of the hydrogel sensor and slightly smaller than that of the whole wireless sensing system. **b** Wearable demonstration of the sensing device. **c** Configuration of the H_2S sensing system consisted of a Zn/Ag/DNO sensor, a customized BLE module, and real-time monitoring App.

Supplementary Tables

The LOD can be expressed as:

$$LOD = \frac{3RMS_{Noise}}{Slope} \quad (1)$$

where RMS_{Noise} is the root mean square deviation of the noise from the dynamic OCV curve and *Slope* is the sensitivity of the sensor here. As for the Zn/Ag/Gly1h-DNO, the sensitivity was experimentally measured to be 25.6 mV/ppm and the RMS_{Noise} was calculated to be 0.006214 mV according to the data in this table. Thus, the theoretical LOD is calculated as 0.73 ppb.

Supplementary Table 1. The 5th order polynomial fitting data used to calculate the LOD of the sensors at the baseline before exposure to H_2S.

**Time (s)**	**V_i-V**	**$(V_i\text{-}V)^2$**
**310**	-0.00376	1.41E-05
**320**	5.43E-03	2.95E-05
**330**	-0.00653	4.26E-05
**340**	0.00325	1.06E-05
**350**	0.00719	5.17E-05
**360**	-0.00432	1.87E-05
**370**	-0.00332	1.1E-05
**380**	0.00912	8.32E-05
**390**	0.00846	7.16E-05
**400**	-0.00382	1.46E-05

Supplementary Table 2. Comparison in the LOD, operating temperature, self-powered capacity, flexibility, maximal strain, and transparency of various H_2S sensors.

Sensing materials	LOD (ppb)	Operating temperature (°C)	Self-powered	Flexible	Maximal Strain	Transparent
**2D $NbWO_6$ [1]**	500	150	No	No	/	No
**WO_3-coated SnO_2 nanowires[2]**	100	200	No	No	/	No
**TiO_2 thin films[3]**	100	100	No	No	/	No
**CuO/In_2O_3 [4]**	200	70	No	No	/	No
**2D MoO_3 nanoflakes[5]**	500	300	No	No	/	No
**Cu-doped ZnO/RGO nanocomposites[6]**	136	24	No	No	/	No
**null-Fe_2O_3 nanoparticles[7]**	50	300	No	No	/	No
**SnO_2/rGO/PANI ternary nanocomposite[8]**	50	25	No	Yes	0%	No
**MOF-5/CS/IL membrane[9]**	1000	25	No	Yes	0%	No
**Co_2SnO_4-SE[10]**	100	510	No	No	/	No
**Mesoporous SnO_2 [11]**	0.5	92	No	No	/	No
**porous $ZnFe_2O_4$ nanosheets[12]**	1000	85	No	No	/	No
**Zn/Ag/DNO[This work]**	0.73	-10~40	Yes	Yes	358%	Yes

Supplementary References

1. Wang, J. et al. Ultrathin 2D $NbWO_6$ Perovskite Semiconductor Based Gas Sensors with Ultrahigh Selectivity under Low Working Temperature. *Adv. Mater.* **34**, 2104958 (2022).
2. Hoa, T. T. N. et al. Highly selective H_2S gas sensor based on WO_3-coated SnO_2 nanowires. *Mater. Today Commun.* **26**, 102094 (2021).
3. Nagmani, Pravarthana, D., Tyagi, A., Jagadale, T. C., Prellier, W., Aswal, D. K. Highly sensitive and selective H_2S gas sensor based on TiO_2 thin films. *Appl. Surf. Sci.* **549**, 149281 (2021).
4. Li, S. et al. Metal-Organic frameworks-derived bamboo-like CuO/In_2O_3 Heterostructure for high-performance H_2S gas sensor with Low operating temperature. *Sens. Actuators B* **310**, 127828 (2020).
5. Bao, J., Zhang, Z., Zheng, Y. H_2S sensor based on two-dimensional MoO_3 nanoflakes: Transition between sulfidation and oxidation. *Sens. Actuators B* **345**, 130408 (2021).
6. Shewale, P. S., Yun, K.-S. Synthesis and characterization of Cu-doped ZnO/RGO nanocomposites for room-temperature H_2S gas sensor. *J. Alloys Compd.* **837**, 155527 (2020).
7. Li, Z. et al. A fast response & recovery H_2S gas sensor based on alpha-Fe_2O_3 nanoparticles with ppb level detection limit. *J. Hazard. Mater.* **300**, 167-174 (2015).
8. Zhang, D., Wu, Z., Zong, X. Flexible and highly sensitive H_2S gas sensor based on in-situ polymerized SnO_2/rGO/PANI ternary nanocomposite with application in halitosis diagnosis. *Sens. Actuators B* **289**, 32-41 (2019).
9. Ali, A., Alzamly, A., Greish, Y. E., Bakiro, M., Nguyen, H. L., Mahmoud, S. T. A Highly Sensitive and Flexible Metal-Organic Framework Polymer-Based H_2S Gas Sensor. *ACS Omega* **6**, 17690-17697 (2021).
10. Wang, C. et al. Mixed potential type H_2S sensor based on stabilized zirconia and a Co_2SnO_4 sensing electrode for halitosis monitoring. *Sens. Actuators B* **321**, 128587 (2020).
11. Song, B.-Y. et al. Highly selective ppb-level H_2S sensor for spendable detection of exhaled biomarker and pork freshness at low temperature: Mesoporous SnO_2 hierarchical architectures derived from waste scallion root. *Sens. Actuators B* **307**, 127662 (2020).
12. Gao, X., Sun, Y., Zhu, C., Li, C., Ouyang, Q., Chen, Y. Highly sensitive and selective H_2S sensor based on porous $ZnFe_2O_4$ nanosheets. *Sens. Actuators B* **246**, 662-672 (2017).
