# Supplementary material for: Design of stretchable and self-powered sensing device for portable and remote trace biomarkers detection
Source: Nat Commun. 2023 Aug 26;14:5221. doi: 10.1038/s41467-023-40953-z (PMC10460451; doi:10.1038/s41467-023-40953-z)
Supplement: Supplementary file 2 — Description of Additional Supplementary Files [file 41467_2023_40953_MOESM2_ESM.pdf]

**Description of Additional Supplementary Files**

File Name: Supplementary Movie 1
Description: Video of hydrogel in tensile strains. The PAM/CA DN hydrogel was stretched to 400% strain and returned to its original length.

File Name: Supplementary Movie 2
Description: Wireless alarm demonstration. A threshold of 650 mV was set in advance. Before exposure to $H_2S$, the recorded voltage stayed at above 720 mV and the phone showed NORMAL in green. When the H2S was on the voltage began to drop. And the phone displayed a red ALARM sign once the voltage fell below the threshold. When the $H_2S$ was off, the voltage rose and returned to a level above the threshold, leading to the reappearance of the green NORMAL sign.

File Name: Supplementary Movie 3
Description: Real-time and remote monitoring demonstration. The sensing device is in the lab, and the researcher is using a tablet in the office to track the OCV of the sensor in real time. Subsequently, 4 ppm of $H_2S$ was released into the test chamber to simulate the H2S leakage in the lab. The phone in the lab immediately showed a rapid decline in the OCV of the sensor, and the researcher found an abnormality through the tablet. When the sensor's OCV dropped below the threshold, the phone and tablet App raised an alarm, and the researcher went to the lab to deal with the $H_2S$ gas leaks issue.
